# ALK‐Positive Histiocytosis With Unilateral Breast Involvement: A Case Report

**DOI:** 10.1002/ccr3.71822

**Published:** 2026-01-16

**Authors:** Xuechun Liu, Dong Ren, Yanfang Liang

**Affiliations:** ^1^ Department of Pathology Dongguan Hospital Affiliated to Jinan University, Binhaiwan Central Hospital of Dongguan Dongguan China; ^2^ Department of Pathology The First Affiliated Hospital of Jinan University Guangzhou China; ^3^ Guangdong Provincial Key Laboratory of Medical Immunology and Molecular Diagnostics Dongguan China; ^4^ Department of Pathology and Laboratory Medicine University of California Irvine California USA

**Keywords:** ALK, breast, histiocytosis, histopathology

## Abstract

APH is a rare disorder characterized by the proliferation of ALK‐expressing histiocytes with variable anatomical involvement; however, mammary involvement is exceptionally rare. A 32‐year‐old woman presented with a painless right breast mass. Ultrasound identified a 9 × 8 mm hypoechoic nodule, categorized as BI‐RADS 4A. Microscopy showed spindle cells in whorled patterns with characteristic nuclear folding. Immunohistochemistry demonstrated positivity for CD68, CD163, and ALK, and negativity for S‐100, CD1a, and CD207. The Ki‐67 proliferation index was approximately 10%. Fluorescence in situ hybridization (FISH) confirmed ALK gene rearrangement (The partner gene not identified), establishing the diagnosis of APH. The patient underwent complete resection without adjuvant therapy, and no recurrence was observed at the 15‐month ultrasound follow‐up. Accurate diagnosis of APH requires integration of histopathology, immunohistochemistry, and molecular testing to distinguish from other histiocytic disorders, with this case highlighting the rare presentation of APH in the breast.

## Key Clinical Message

ALK‐positive histiocytosis (APH) is rare in the breast. Diagnosis requires histopathology showing spindle cells, CD68, CD163, and ALK‐positive immunohistochemistry excluding CD1a and CD207, and confirmed ALK rearrangement. No recurrence was achieved with complete surgical resection alone, pointing to an indolent clinical course in this case of localized mammary APH, but calls for further validation.

## 1 Introduction

APH is a rare disorder characterized by the proliferation of morphologically distinctive histiocytes expressing ALK protein. Since its initial description by Chan et al. in 2008 [1], numerous cases have been reported worldwide. The *2022 WHO Classification of Haematolymphoid Tumors* now formally recognizes APH as a distinct entity [2]. Although APH can involve various anatomical sites—including the lung, skin, and central nervous system [3, 4, 5, 6, 7], mammary involvement remains exceptionally rare(Table 1). This report presents a rare case of mammary APH and summarizes its clinicopathological features to aid in the recognition and understanding of this uncommon entity.

**TABLE 1:** Summary of clinical characteristics and outcomes in published cases of mammary ALK‐positive histiocytosis.

First author, year	Age (years)	Ethnicity	Tumor size (mm)	ALK fusion partner	Treatment	Follow‐up (months)
Osako, 2022 [8]	38	Japanese	17	KIF5B (24)‐ALK (20)	Resection	No evidence of disease (11 months)
Osako, 2022 [8]	45	Japanese	13	KIF5B (24)‐ALK (20)	Resection	No evidence of disease (13 months)
Zhou, 2023 [9]	46	Hispanic	20	KIF5B‐ALK	Crizotinib	No evidence of disease (4 months)
Kurita, 2022 [10]	38	NA	12	KIF5B‐ALK	NA	NA
Chang, 2019 [11]	40	Chinese	NA	KIF5B‐ALK	Resection	No evidence of disease (42 months)
Kashima J, 2021 [12]	45	Asian	16	KIF5BALK	Resection	No evidence of disease (1 months)
Kashima, 2021 [12]	16	Asian	21	KIF5BALK	Resection, alectinib	No evidence of disease (44 months)
The present case	32	Chinese	9	ALK gene rearrangement(FISH)	Resection	No evidence of disease (15 months)

## 2 Case Presentation

**FIGURE 1:**
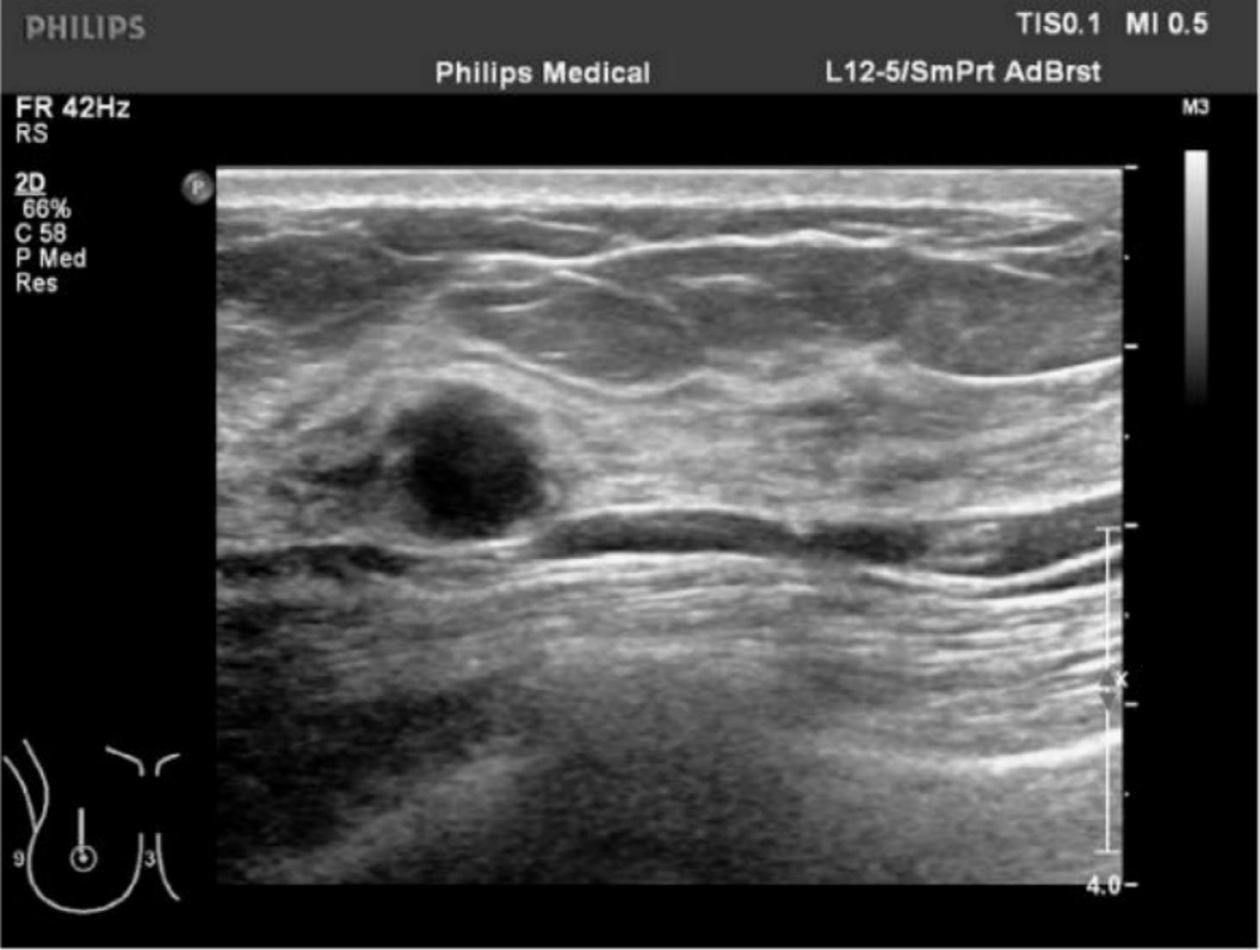
Ultrasound findings: A hypoechoic nodule in the right breast (BI‐RADS category 4A).

**FIGURE 2:**
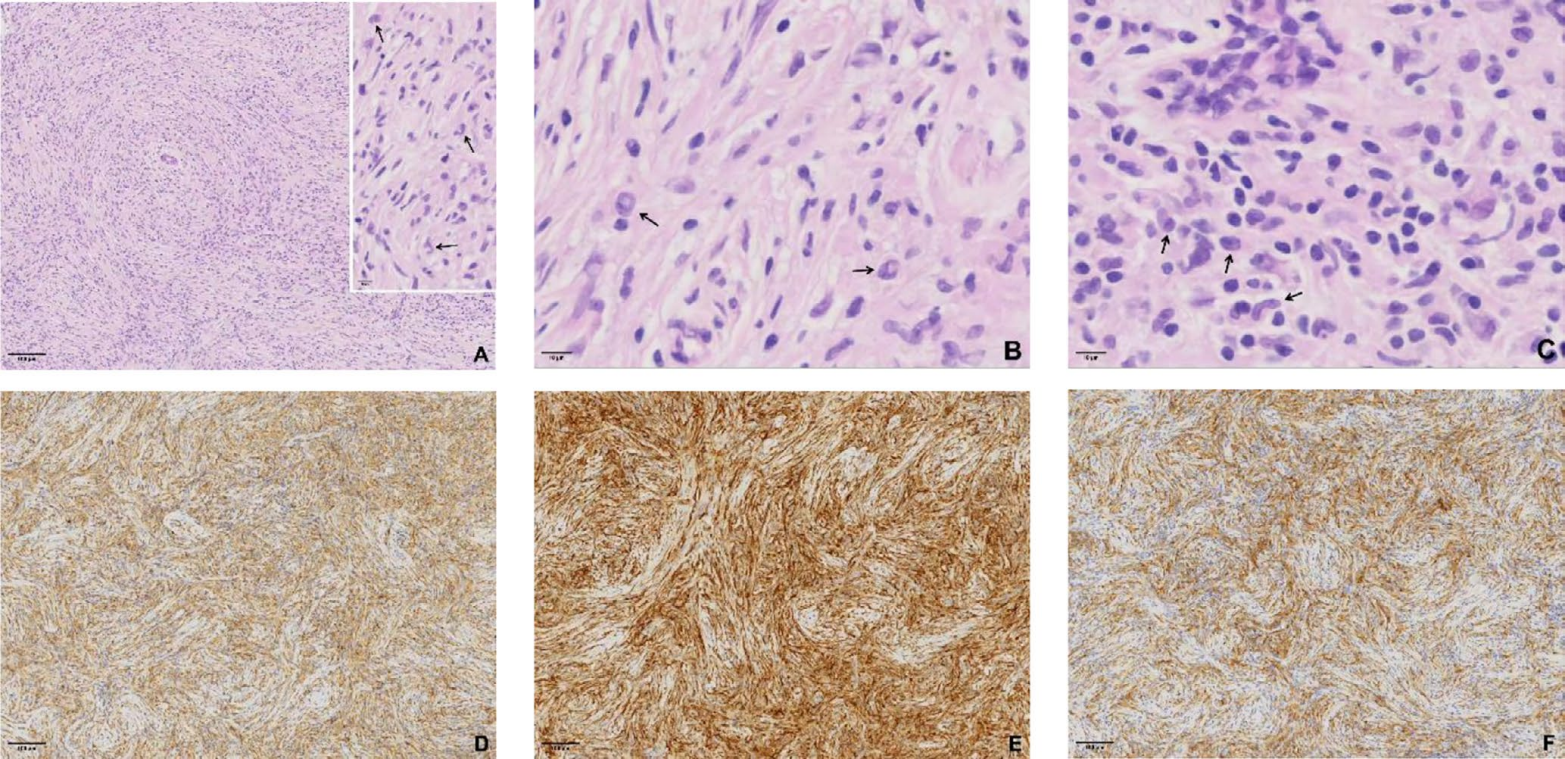
Histologic features of ALK‐positive histiocytosis. (A) Spindle‐shaped cells arranged in whorled patterns (HE, ×40). Horseshoe‐shaped and bean‐like or reniform folding nuclei (→, HE, ×400). (B) Doughnut‐shaped histiocytic nuclei (→, HE, ×400). (C) Characteristic nuclear folding and nuclear grooves (→, HE, ×400). (D) CD68 cytoplasmic positivity (EnVision method, ×40). (E) CD163 cytoplasmic positivity (EnVision method, ×40). (F) ALK cytoplasmic positivity (EnVision method, ×40).

**FIGURE 3:**
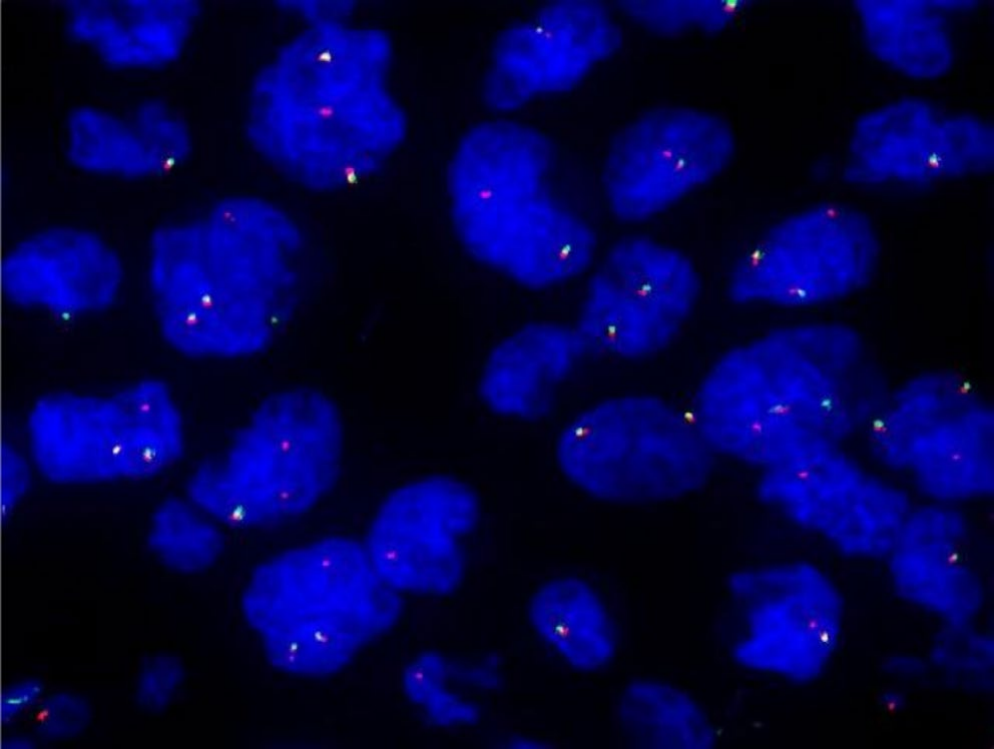
ALK gene rearrangement confirmed by FISH.

A 32‐year‐old woman presented in December 2023 with a painless right breast mass. Physical examination revealed no erythema, swelling, ulceration, or skin changes in the breast and no abnormalities at other sites. Laboratory tests including complete blood count and biochemistry were within normal limits. The patient underwent breast ultrasound and pathological examination, with no systemic staging imaging performed. Breast ultrasound identified a 9 × 8 mm hypoechoic nodule at the 12 o'clock position, located 33 mm from the nipple. The lesion exhibited heterogeneous echogenicity, circumscribed margins, an aspect ratio greater than 1, and no detectable vascularity (CDFI Grade 0). It was classified as BI‐RADS 4A (Figure 1). Gross examination of the excised tissue revealed fragmented grayish‐white to yellow material measuring 20 × 20 × 20 mm (Adjacent normal tissue included via a Mammotome biopsy). Histologic evaluation showed spindle cells arranged in whorled patterns, with abundant pale cytoplasm and vesicular nuclei featuring characteristic doughnut‐shaped or reniform folding (Figure 2). Additional findings included Touton giant cells, focal inflammatory infiltrates, and stromal hyperplasia. Immunohistochemistry demonstrated positivity for CD68, CD163, and ALK, and negativity for S‐100, CD1a, and CD207. The Ki‐67 proliferation index was approximately 10% (Figure 2). FISH confirmed ALK gene rearrangement (Figure 3), establishing the diagnosis of APH.

All tissue samples were fixed in 10% neutral buffered formalin, embedded in paraffin, and sectioned into 4 μm slices for histological examination by hematoxylin–eosin (HE) staining and immunohistochemical evaluation of tumor cell immunophenotypes. ALK monoclonal antibody and Ki‐67 detection were performed by Gene Biotechnology, while CD68, CD163, Calponin, Desmin, SMA, P‐CK, S‐100, CD34, CD1a, and CD207 were analyzed by Maixin Biotechnology. ALK gene rearrangements were detected by FISH using probes from Abbott Gene Tech (Shanghai).

## 3 Differential Diagnosis, Investigations and Treatment

Histiocytes in APH typically express histiocytic markers such as CD68, CD163, lysozyme, and ALK. They lack expression of CD1a and CD207. However, a reported case of APH was positive for S100 and dendritic cell markers (fascin, Factor XIIIa), yet still negative for both CD1a and Langerin [1]. The differential diagnosis of APH includes other histiocytic disorders, most notably Erdheim‐Chester disease (ECD), Langerhans cell histiocytosis (LCH), and Rosai‐Dorfman disease (RDD).

ECD typically affects patients aged 55–60 and involves the bones, cardiovascular system, and retroperitoneum. It is characterized by foamy mononuclear histiocytes with small nuclei, frequent multinucleated or Touton‐type giant cells, and variable fibrosis. Over 50% of ECD cases harbor BRAF V600E mutations [11]. In contrast to APH, ECD features foamy histiocytes and lacks ALK rearrangements, although rare ALK expression without rearrangement has been reported [12].

LCH usually presents with localized bone pain or tumor‐like lesions and is characterized by cells resembling Langerhans cells that are actually myeloid precursors sharing their immunophenotype [13]. These cells are positive for CD1a, CD207, and S100, and contain pathognomonic Birbeck granules on ultrastructural analysis. Approximately 50% of LCH cases also carry BRAF V600E mutations, differing from APH, which consistently shows ALK rearrangements and is negative for CD1a and CD207 [14].

RDD is characterized by sinusoidal expansion by large histiocytes with abundant cytoplasm, prominent nucleoli, and the presence of emperipolesis. RDD cells are positive for S100, CD163, and CD68, but negative for CD1a [15]. Unlike the sinusoidal growth pattern of RDD, mammary APH typically demonstrates a spindle cell morphology, aiding in its distinction.

Spindle cell morphology in APH may also mimic inflammatory myofibroblastic tumor (IMT), which expresses SMA and desmin but lacks histiocytic markers. Although around 50% of IMTs exhibit ALK expression, they often harbor TPM3‐ALK, TPM4‐ALK, or CLTC‐ALK fusions, distinct from the KIF5B‐ALK fusion predominantly seen in APH [11].

The patient in this case underwent complete surgical resection without adjuvant therapy. ALK inhibitors represent the primary treatment modality for APH, often achieving durable clinical remission, although consensus on their use as first‐line versus for refractory cases remains lacking. Notably, a reported case of ALK‐positive ECD‐like histiocytosis arising in the setting of chronic lymphocytic leukemia/small lymphocytic lymphoma (CLL/SLL) demonstrated a favorable response to the Bruton tyrosine kinase (BTK) inhibitor ibrutinib, suggesting potential therapeutic utility of BTK inhibition in select cases [16]. Molecular profiling remains essential for guiding targeted therapy and optimizing individualized treatment strategies.

## 4 Conclusions and Results (Outcome and Follow‐Up)

The patient showed no evidence of recurrence at the 15‐month ultrasound follow‐up. APH is generally associated with a favorable prognosis, with most patients achieving stable disease or complete remission following systemic chemotherapy, surgical resection, or targeted therapy. However, certain cases may exhibit aggressive histological features—for example, a reported mesenteric case demonstrated intravascular invasion involving large veins [17]. Due to the rarity of APH, the correlation between histologic features and clinical behavior remains poorly defined, underscoring the need for further investigation. Additionally, treatment‐related complications may significantly impact outcomes and warrant careful management. In the present case, serial ultrasound examinations confirmed disease stability without evidence of progression or metastasis, in line with the overall indolent biological behavior of this tumor.

## 5 Discussion

APH presents notable diagnostic challenges, with multiple cases reported across Asian populations, particularly in China and Japan, spanning a broad age range from 2 months to 66 years. This entity most commonly involves the central nervous system, skin, and lungs, while mammary involvement remains exceptionally rare. Current evidence suggests that mammary APH primarily affects Asian females aged 30–40 years, though no clear clinicopathological definition has been established to date [8]. Cutaneous presentations typically appear as papules or nodules, in contrast to mammary lesions, which are often asymptomatic and incidentally detected on imaging, as demonstrated in the present case.

Histopathological features of APH demonstrate considerable morphological diversity. It is typically composed of large histiocytes with abundant eosinophilic cytoplasm and irregular nuclei characterized by delicate chromatin and complex nuclear folding patterns, distinguishing it from other histiocytic proliferations. In some cases, the lesion comprises uniform spindle cells arranged in fascicular or storiform patterns, often accompanied by lymphocytic infiltration, which can mimic inflammatory disorders or proliferative lesions such as inflammatory myofibroblastic tumor (IMT). Notably, spindle cell morphology is frequently observed in mammary APH, while it remains relatively uncommon in APH at other anatomical sites [8]. Our case is consistent with previously reported features of mammary APH, including prominent spindle cell proliferation, with subtle histological variation across anatomical sites as summarized in recent literature (Table 2).

**TABLE 2:** Histopathological features of APH in different anatomical sites.

Case	Sex	Age	Affected site	Histopathological features
1	Male	2 days postnatal	Liver	Sinusoidal infiltration pattern with compression of adjacent hepatocytes and variable portal tract involvement [4].
2	Female	7 and 10 years old	Cerebellar vermis, central cortex	Sheets of large epithelioid cells with irregularly folded nuclei, fine chromatin, foamy cells, Touton giant cells, and focal emperipolesis [7].
3	Male	38 years	Mediastinum	Large histiocytes with abundant eosinophilic cytoplasm, cleaved or lobulated nuclei, and small nucleoli (meningothelial‐like morphology). Scattered foamy cells, small lymphocytes, rare Touton giant cells, and emperipolesis [3].
4	Male	49 years	Lymph node	Polygonal histiocytes (medium to large) with moderate eosinophilic cytoplasm, fine chromatin, inconspicuous nucleoli, and frequent nuclear grooves or folds. Focal pleomorphic cells with prominent nucleoli and scattered Touton giant cells [5].
5	Female	52 years	Lung	Round to spindle‐shaped cells with abundant eosinophilic vesicular cytoplasm, mild nuclear atypia, and indistinct nucleoli. Background of foamy histiocytes, lymphocytes, and hemosiderin deposits [6].

Recent molecular advances have begun to elucidate the genomic landscape of histiocytic disorders. ALK gene rearrangement is considered the defining molecular hallmark of APH and a potential oncogenic driver. While ALK fusions occur with various partners across tumors, specific fusion partners tend to be enriched in particular disease entities. In APH, KIF5B‐ALK is the most frequently reported fusion, though rare variants include *TPM3‐ALK, COL1A2‐ALK, TRIM33‐ALK, CLTC‐ALK, TFG‐ALK, DCTN1‐ALK*, and *EML4‐ALK* [3].

*KIF5B‐ALK*–positive cases typically display sheet‐like histiocytic growth composed of cells with abundant eosinophilic cytoplasm, irregular nuclear contours (including folding, lobulation, and clefting), finely dispersed chromatin, and inconspicuous nucleoli.

*TRIM33(exon12)‐ALK(exon20)* fusions are associated with nested histiocytic proliferation within a delicate capillary network, occasionally exhibiting hemangiopericytoma‐like vascular patterns. Histiocytes show indistinct cytoplasmic borders, moderately eosinophilic cytoplasm, and ovoid nuclei arranged in alveolar patterns, with prominent basophilic nucleoli and no nuclear grooves. Mammary APH frequently demonstrates lymphoid follicles with plasma cells, eosinophils, and lymphocytes, contributing to IMT‐like histologic features [17].

*COL1A2(exon51)‐ALK(exon19)* fusions are typically associated with cutaneous or soft tissue lesions, characterized by distinct histiocytic islands rather than sheets. The neoplastic cells exhibit amphophilic cytoplasm and monotonous nuclei with single prominent nucleoli [17].

## Author Contributions

**Xuechun Liu:** conceptualization, data curation, investigation, project administration, visualization, writing – original draft, writing – review and editing. **Dong Ren:** writing – review and editing. **Yanfang Liang:** funding acquisition, supervision, writing – review and editing.

## Funding

This work was supported by the Guangdong Basic and Applied Basic Research Foundation, 2021B1515140066. High Level Scientific Research Incubation Foundation of Binhaiwan Central Hospital of Dongguan, 2024002.

## Consent

The patient provided written informed consent for the publication of this case report, in accordance with the journal's consent policy.

## Conflicts of Interest

The authors declare no conflicts of interest.

## Data Availability

The data used to support the findings of this study are included in the article.

## References

[1] J. K. Chan , L. Lamant , E. Algar , et al., “ALK+ Histiocytosis: A Novel Type of Systemic Histiocytic Proliferative Disorder of Early Infancy,” Blood 112, no. 7 (2008): 2965–2968, 10.1182/blood-2008-03-147017. 18660380

[2] J. D. Khoury , E. Solary , O. Abla , et al., “The 5th Edition of the World Health Organization Classification of Haematolymphoid Tumours: Myeloid and Histiocytic/Dendritic Neoplasms,” Leukemia 36, no. 7 (2022): 1703–1719, 10.1038/s41375-022-01613-1. 35732831 PMC9252913

[3] W. Liu , H. J. Liu , W. Y. Wang , et al., “Multisystem ALK‐Positive Histiocytosis: A Multi‐Case Study and Literature Review,” Orphanet Journal of Rare Diseases 18, no. 1 (2023): 53, 10.1186/s13023-023-02649-x. 36915094 PMC10010018

[4] H. Huang , G. Gheorghe , P. E. North , and M. Suchi , “Expanding the Phenotype of ALK‐Positive Histiocytosis: A Report of 2 Cases,” Pediatric and Developmental Pathology 21, no. 5 (2018): 449–455, 10.1177/1093526617740784. 29224419

[5] L. Qiu , S. P. Weitzman , L. J. Nastoupil , M. D. Williams , L. J. Medeiros , and F. Vega , “Disseminated ALK‐Positive Histiocytosis With *KIF5B‐ALK* Fusion in an Adult,” Leukemia & Lymphoma 62, no. 5 (2021): 1234–1238, 10.1080/10428194.2020.1861273. 33353436

[6] Y. Bai , W. Sun , D. Niu , et al., “Localized ALK‐Positive Histiocytosis in a Chinese Woman: Report of a Case in the Lung With a Novel EML4‐ALK Rearrangement,” Virchows Archiv 479, no. 6 (2021): 1079–1083, 10.1007/s00428-021-03092-8. 33825946

[7] C. G. Lucas , A. Gilani , D. A. Solomon , et al., “ALK‐Positive Histiocytosis With KIF5B‐ALK Fusion in the Central Nervous System,” Acta Neuropathologica 138, no. 2 (2019): 335–337, 10.1007/s00401-019-02027-7. 31119374 PMC6712982

[8] T. Osako , A. Kurisaki‐Arakawa , A. Dobashi , et al., “Distinct Clinicopathologic Features and Possible P‐Athogenesis of Localized ALK‐Positive Histiocytosis of the Breast,” American Journal of Surgical Pathology 46, no. 3 (2022): 344–352, 10.1097/PAS.0000000000001794. 34482333

[9] Y. Zhou , M. Hurtado‐Castillo , and O. Pandey , “Case Report: ALK‐Positive Histiocytosis Presented as Bilateral Synchronous Breast Masses With Long‐Term Remission on Crizotinib,” Frontiers in Medicine 10 (2023): 1288849, 10.3389/fmed.2023.1288849. 38093982 PMC10716452

[10] A. Kurita , M. Yoshida , T. Murata , A. Yoshida , N. Uchiyama , and S. Takayama , “A Case of ALK‐Positive Histiocytosis With Multiple Lesions in the Unilateral Breast: A Case Report,” International Journal of Surgery Case Reports 97 (2022): 107435, 10.1016/j.ijscr.2022.107435. 35908452 PMC9403183

[11] K. T. E. Chang , A. Z. E. Tay , C. H. Kuick , et al., “ALK‐Positive Histiocytosis: An Expanded Clinicopathologic Spectrum and Frequent Presence of KIF5B‐ALK Fusion,” Modern Pathology 32, no. 5 (2019): 598–608, 10.1038/s41379-018-0168-6. 30573850

[12] J. Kashima , M. Yoshida , K. Jimbo , et al., “ALK‐Positive Histiocytosis of the Breast: A Clinicopatholo‐Gic Study Highlighting Spindle Cell Histology,” American Journal of Surgical Pathology 45, no. 3 (2021): 347–355, 10.1097/PAS.0000000000001567. 32826530

[13] C. E. Allen , L. Li , T. L. Peters , et al., “Cell‐Specific Gene Expression in Langerhans Cell Histiocytosis Lesi‐Ons Reveals a Distinct Profile Compared With Epidermal Langerhans Cells,” Journal of Immunology 184, no. 8 (2010): 4557–4567, 10.4049/jimmunol.0902336. PMC3142675 20220088

[14] C. Rodriguez‐Galindo and C. E. Allen , “Langerhans Cell Histiocytosis,” Blood, the Journal of the American Society of Hematology 135 (2020): 1319–1331, 10.1182/blood.2019000934. 32106306

[15] J. C. Hoffmann , C. Y. Lin , S. Bhattacharyya , et al., “Rosai‐Dorfman Disease of the Breast With Variable IgG4+ Plasma Cells: A Diagnostic Mimicker of Other Malignant and Reactive Entities,” American Journal of Surgical Pathology 43, no. 12 (2019): 1653–1660, 10.1097/PAS.0000000000001347. 31436555

[16] C. Syrykh , L. Ysebaert , S. Péricart , et al., “ALK‐Positive Histiocytosis Associated With Chronic Lymphoc‐Ytic Leukaemia/Small Lymphocytic Lymphoma: A Multitarget Response Under Ibrutinib,” Virchows Archiv 478, no. 4 (2021): 779–783, 10.1007/s00428-020-02937-y. 33011863

[17] T. A. N. Tran , K. T. E. Chang , C. H. Kuick , J. Y. Goh , and C. C. Chang , “Local ALK‐Positive Histiocytosis With U‐Nusual Morphology and Novel *TRIM33‐ALK* Gene Fusion,” International Journal of Surgical Pathology 29, no. 5 (2021): 543–549, 10.1177/1066896920976862. 33243034

